# High-Strength Controllable Resin Plugging Agent and Its Performance Evaluation for Fractured Formation

**DOI:** 10.3390/gels10080511

**Published:** 2024-08-02

**Authors:** Xiongwei Liu, Biao Qi, Xiuping Chen, Ziyao Shen, Jingbin Yang

**Affiliations:** 1 Key Laboratory of Enhanced Oil Recovery in Carbonate Fractured-Vuggy Reservoirs, SINOPEC, Xinjiang 830011, China; 2 SINOPEC Northwest Company of China Petroleum and Chemical Corporation, Xinjiang 830011, China; 3 School of Petroleum Engineering, China University of Petroleum (East China), Qingdao 266580, China

**Keywords:** fractured formation, high strength, resin plugging agent, curing effect, plugging performance

## Abstract

Lost circulation is a common and complicated situation in drilling engineering. Serious lost circulation may lead to pressure drop in the well, affect normal drilling operations, and even cause wellbore instability, formation fluid flooding into the wellbore, and blowout. Therefore, appropriate preventive and treatment measures need to be taken to ensure the safe and smooth operation of drilling operations. So, it is necessary to conduct in-depth research on the development and performance of the plugging materials. In this study, urea formaldehyde resin with high temperature resistance and strength was used as the main raw material, and the curing conditions were optimized and adjusted by adding a variety of additives. The curing time, compressive strength, temperature resistance, and other key performance indexes of the resin plugging agent were studied, and a resin plugging agent system with excellent plugging performance was prepared. The formula is as follows: 25% urea formaldehyde resin +1% betaine +1% silane coupling agent KH-570 + 3% ammonium chloride +1% hexamethylenetetramine +1% sodium carboxymethyl cellulose. The optimal curing temperature is between 60 and 80 °C, with a controllable curing time of 1–3 h. Experimental studies examined the rheological and curing properties of the resin plugging agent system. The results showed that the viscosity of the high-strength curable resin system before curing remained stable with increasing shear rates. Additionally, the storage modulus and loss modulus of the resin solutions increased with shear stress, with the loss modulus being greater than the storage modulus, indicating a viscous fluid. The study also investigated the effect of different salt ion concentrations on the curing effect of the resin plugging system. The results showed that formation water containing Na^+^ at concentrations between 500 mg/L and 10,000 mg/L increased the resin’s curing strength and reduced curing time. However, excessively high concentrations at lower temperatures reduced the curing strength. Formation water containing Ca^2+^ increased the curing time of the resin plugging system and significantly impacted the curing strength, reducing it to some extent. Moreover, the high-strength curable resin plugging agent system can effectively stay in various fracture types (parallel, wedge-shaped) and different fracture sizes, forming a high-strength consolidation under certain temperature conditions for effective plugging. In wedge-shaped fractures with a width of 10 mm, the breakthrough pressure of the high-strength curable resin plugging agent system reached 8.1 MPa. As the fracture width decreases, the breakthrough pressure increases, reaching 9.98 MPa in wedge-shaped fractures with an outlet fracture width of 3 mm, forming a high-strength plugging layer. This research provides new ideas and methods for solving drilling fluid loss in fractured loss zones and has certain application and promotion value.

## 1. Introduction

As oil and gas field exploration and development extend to deeper layers, lost circulation becomes increasingly sudden and complex. Although plugging technology has significantly improved the success rate of plugging in recent years, the effectiveness of plugging in highly permeable reservoirs remains inadequate, especially in formations with large fractures and developed karst caves [1,2]. Therefore, it is necessary to conduct in-depth research on the mechanisms of drilling fluid loss and plugging, the high-temperature and high-pressure stability of plugging materials, and their retention and filling characteristics [3]. Currently, common plugging materials include bridging agents, high-fluid-loss agents, and gels, which can effectively prevent drilling fluid loss and improve the success rate of plugging [4,5,6,7]. However, under deep high-temperature and high-pressure conditions, the success rate of single-time plugging is not ideal, and these materials are unsuitable for reservoirs with large fractures and karst caves [8,9]. The resin plugging material has excellent properties, such as heat resistance, pressure resistance, and non-flammability [10,11]. It is used as a single plugging material, or the resin material is coated onto the surface of the bridge material, such as mica, fiber, walnut shell, and quartz sand. Under the influence of the wall temperature, the consolidation strength increases, and the plugging success rate can be effectively increased.

The thermosetting resin will form a hardened structure with a cross-linked network structure during curing [12]. It is characterized by high temperature resistance and high pressure resistance. It is not easy to burn, and the product size is stable, but it is brittle. Currently, there are mainly unsaturated polyester resin, epoxy resin, phenolic resin, urea formaldehyde resin, and other thermosetting resins [13,14,15]. Jiang et al. prepared SA/MPF-E44 composite microcapsule material with sodium alginate/melamine phenolic resin as shell and epoxy resin as the core using an in situ polymerization method [16]. The modified melamine phenolic resin has excellent mechanical and thermal stability and is a good self-healing material. Sousa et al. modified unsaturated polyester resin with nano-Al_2_O_3_ and ZrO_2_ particles, resulting in a composite material with significantly enhanced mechanical properties [17]. Traditional cement slurry plugging materials are composed of gypsum, silicate, cement, and lime. Due to the large proportion of cement, its hardness after solidification is generally higher than the hardness of the formation [18,19]. When re-drilling, new holes are likely to be formed, resulting in the abandonment of the old hole. In addition, cement slurry will also cause pollution to the drilling fluid, resulting in drilling fluid failure [20]. At this time, a layer of resin is coated on the surface of mica, fiber, walnut shell, quartz sand, and other materials so that it will aggregate after reaching the lost layer, forming a consolidation, which can effectively enhance the strength of the plugging layer and improve the success rate of a plug.

Thermosetting resin material can adapt to different formation temperatures and pressures because of its own kind of richness and excellent mechanical properties, including heat resistance and chemical resistance after modification; it has great potential in the field of oil drilling and production engineering [21,22,23]. Li et al. prepared a butadiene resin/nano-SiO_2_ composite material through continuous emulsion polymerization and used it as a plugging agent for oil-based drilling fluid to improve the plugging efficiency of shale formation [24]. The resin plugging agent can enter the nanopores of the shale formation, significantly reducing fluid intrusion and improving wellbore stability. Huang et al. synthesized a kind of nano-acrylic resin/nano-SiO_2_ composite material with a core–shell structure for water-based drilling fluid, which can improve the plugging efficiency of shale pores, reduce fluid intrusion, and improve wellbore stability during shale gas drilling [25]. Liu et al. prepared nano SiO_2_ modified epoxy resin material using the in situ polymerization method [26] and incorporated it into the cement injection material, which shortened the setting time of the composite slurry, improved the stability of the slurry, realized the delayed effect on cement hydration, and improved the early compressive strength of the composite slurry.

In order to solve the problems of poor downhole cross-linking controllability and low curing strength of commonly used plugging materials, Liu et al. developed a partially etherified amino resin [27]. The resin can be cured within 10 h at a temperature of 80 °C and 130 °C, and it is resistant to up to 10% water-based drilling fluid contamination. After curing at 80 °C for 24 h, the compressive strength of the resin can reach up to 56 MPa, which has a certain application prospect in malignant lost circulation in fractured formation. Batista et al. used polyester resin modified by polyethylene terephthalate as a plugging material, which has high compressive strength and low viscosity and presents a promising application prospect in plugging abandoned wells and remedial operations [12]. Lv et al. prepared an underwater high-temperature and slow-curing epoxy resin plugging system, which can easily pass through simulated formation fractures and solidify at 120 °C [28]. The cured epoxy resin has good compressive strength and can effectively plug fractures, thus being used in drilling fluid plugging in the process of oil and gas drilling. Guo et al. prepared a resin plugging material that can be used for in situ plugging with epoxy resin E-51 as the main material [29]. Compared with conventional plugging materials, epoxy-resin-based plugging materials have good high-temperature and high-pressure resistance, so they can be used as a kind of plugging material with high application potential. Knudsen et al. prepared a thermosetting resin plugging agent to successfully deal with heavy oil mud loss in offshore gas wells in the Middle East [30]. Xu et al. introduced a new plugging technology where resin polycondensation occurs under the action of the bottom hole temperature to consolidate each other, thus increasing the strength and stability of the plugging layer, which has been applied in well G21X3 in Jidong Oilfield; the lost circulation has been plugged successfully [31]. Wang et al. used phenolic resin as the primary material of a high-strength synthetic resin plugging agent combined with other conventional plugging materials to plug the Permian strata in Tahe Oilfield, resulting in a good plugging effect and shortening of the plugging time compared with adjacent wells, achieving excellent and fast drilling [32].

The resin plugging agent in this study has a dense three-dimensional network structure, which can effectively increase toughness and strength, and has good thermal stability and rheology, which can effectively reside in different crack types (parallel, wedge) and different crack sizes and form a high-strength consolidated body under certain temperature conditions for effective plugging. Combined with the application characteristics of high deformation ability before curing and high strength after curing, it can be predicted that the thermosetting resin has a broad application prospect in the field of plugging of fractured-formation drilling fluids. Therefore, thermosetting urea formaldehyde resin with high heat resistance and strength was used as the main raw material in this study. By adding a variety of additives to optimize and adjust the composition ratio and curing conditions, the curing time, compressive strength, temperature resistance, and other key performance indicators of the resin plugging agent were studied. A resin plugging agent system with excellent plugging performance was prepared, and its chemical structure was characterized. At the same time, combined with the rheological and curing characteristics of the resin plugging system and the plugging effect of pressure on fractures of different scales, the plugging mechanism of the resin plugging system was explored, which laid a theoretical foundation for the wide application of thermosetting resin plugging materials.

## 2. Results and Discussion

### 2.1. Preparation of Curable Resin Plugging Agent

#### 2.1.1. Optimization of Resin Plugging Agent Formulations

In order to reduce the cost and increase the curing strength of the resin-consolidated body, a single variable was controlled through an orthogonal experiment, and the added amount of resin, modifier, flow pattern regulator, and curing agent was changed to optimize the optimal formula of the resin plugging system. The curing conditions were studied at 40 °C, 60 °C, 80 °C, and 100 °C, respectively.

##### Optimization of Urea Formaldehyde Resin Concentration

**Figure 1:**
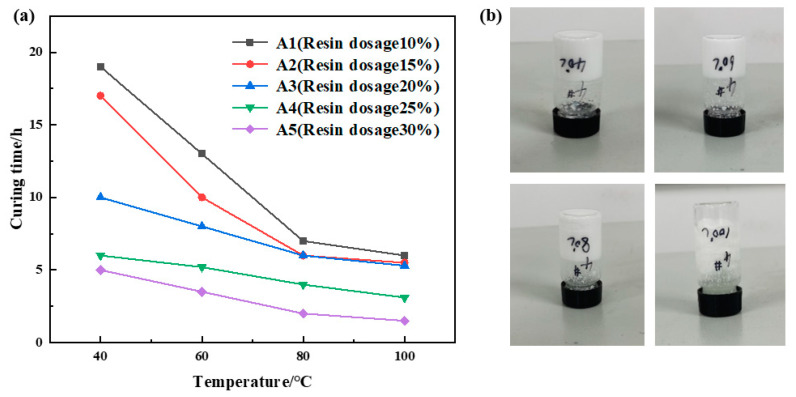
Urea formaldehyde resin concentration optimization results: (**a**) variation in curing time with temperature for different urea formaldehyde resin dosages; (**b**) curing effect of urea formaldehyde resin at 25% addition rate.

The urea formaldehyde resin dosage varied between 10% and 30%, with betaine at 1%, silane coupling agent KH-570 at 1%, ammonium chloride at 5%, hexamethylenetetramine at 2%, sodium carboxymethylcellulose at 1%, and the remaining portion comprising deionized water. The samples underwent curing at temperatures of 40 °C, 60 °C, 80 °C, and 100 °C, respectively. The curing temperature and time for various samples were recorded, and the experimental results are depicted in Figure 1. The experimental results indicate a significant decrease in curing time with increasing temperature at a constant urea formaldehyde resin dosage. However, beyond 80 °C, the reduction in curing time becomes less pronounced. At a constant curing temperature, the curing time decreases with increasing urea formaldehyde resin dosage. A notable decrease in curing time is observed when the resin dosage ranges from 10% to 25%, with a slight decrease when the dosage exceeds 25%. Comprehensive analysis of the experimental results reveals that samples A4 and A5 exhibit short curing times and high strength. Considering resin concentration and cost, it was determined that the optimal urea formaldehyde resin dosage was 25%.

##### Optimization of Betaine Concentration

**Figure 2:**
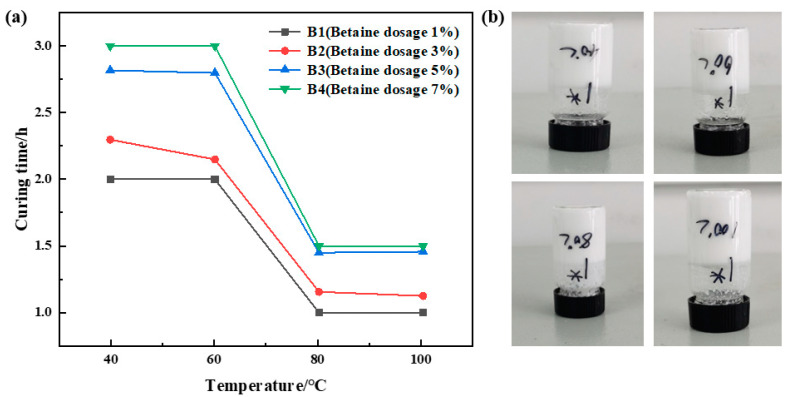
Optimization results of betaine concentration: (**a**) variation in curing time with temperature at different betaine dosages; (**b**) curing effect of betaine dosage of 1%.

The urea formaldehyde resin dosage was 25%, the betaine dosage varied between 1%, 3%, 5%, and 7%, the silane coupling agent KH-570 dosage was 1%, the ammonium chloride dosage was 5%, the hexamethylene tetramine dosage was 2%, and the sodium carboxymethyl cellulose dosage was 1%, with the remaining portion comprising deionized water. Curing of the samples was conducted at temperatures of 40 °C, 60 °C, 80 °C, and 100 °C. The curing temperature and time for different samples were recorded, and the experimental results are depicted in Figure 2. The experimental results indicate a gradual decrease in curing time with increasing temperature at a constant amount of betaine addition. The curing time remains almost constant when the curing temperature exceeds 80 °C. At a constant curing temperature, curing time exhibits a gradual increase with higher amounts of betaine addition. Consequently, higher betaine content prolongs resin curing time, albeit with a slight reduction in curing strength. Considering the experimental outcomes, samples B1 and B2 exhibited superior strength after curing. Therefore, a betaine dosage of 1% was deemed optimal.

##### Optimization of Coupling Agent Concentration

**Figure 3:**
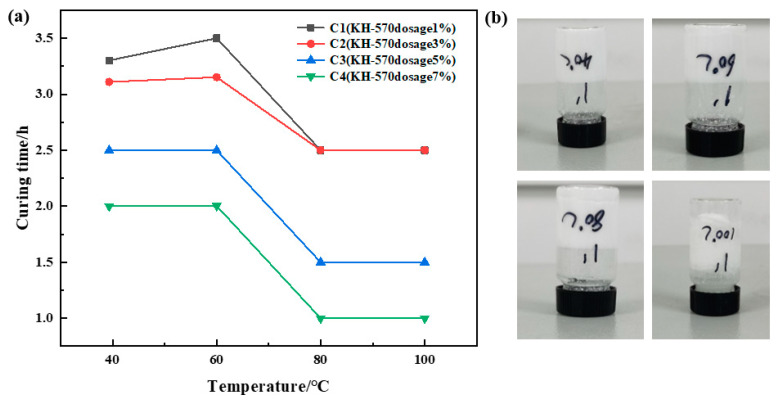
Optimization results of coupling agent concentration: (**a**) variation in curing time with temperature for different coupling agent dosages; (**b**) curing effect of 1% dosage of coupling agent KH-570.

Urea formaldehyde resin was added at a ratio of 25%, betaine at 1%, silane coupling agent KH-570 at ratios of 1%, 3%, 5%, and 7%, ammonium chloride at 5%, hexamethylenetetetramine at 2%, and sodium carboxymethyl cellulose at 1%. The samples underwent curing at temperatures of 40 °C, 60 °C, 80 °C, and 100 °C, respectively. The curing temperature and time were recorded for different samples, and the experimental results are depicted in Figure 3. It was observed that the curing time decreased gradually with increasing temperature at a constant coupling agent dosage. However, the curing time remained almost unchanged when the temperature exceeded 80 °C. At a constant temperature, the change in curing time was not significant with increasing coupling agent dosage, albeit resulting in a slight decrease in the resin’s curing strength. Comprehensive analysis of the experiments revealed that C1 and C2 exhibited superior curing strength. Therefore, it was determined that the optimal dosage of silane coupling agent KH-570 is 1%.

##### Optimization of Ammonium Chloride Concentration

**Figure 4:**
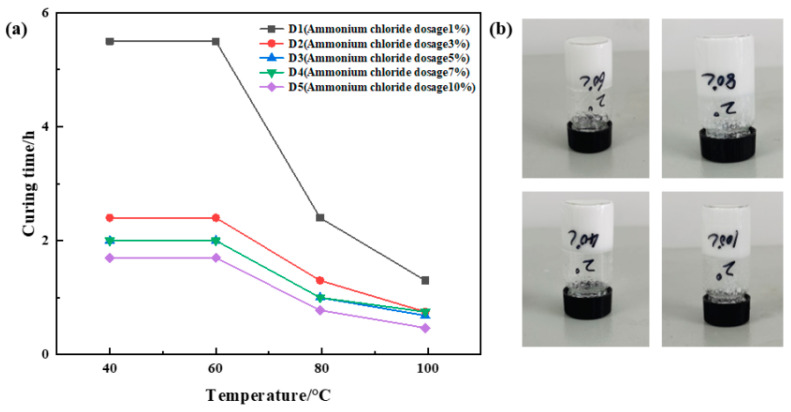
Optimization results of ammonium chloride concentration: (**a**) variation in curing time with temperature at different ammonium chloride dosages; (**b**) curing effect of ammonium chloride addition of 3%.

Urea formaldehyde resin was added at a ratio of 25%, betaine at 1%, silane coupling agent KH-570 at 1%, ammonium chloride at ratios of 1%, 3%, 5%, 7%, and 10%, hexamethylenetetramine at 2%, and sodium carboxymethylcellulose at 1%, with the remainder being deionized water. The samples underwent curing at temperatures of 40 °C, 60 °C, 80 °C, and 100 °C, respectively. The curing temperature and time for different samples were recorded, and the experimental results are depicted in Figure 4. The results showed that the curing time decreased significantly with the increase in temperature under the condition of constant ammonium chloride dosage. Under the condition of constant temperature, the higher the amount of ammonium chloride, the shorter the curing time. However, when the amount of ammonium chloride is greater than 5%, the curing time does not decrease significantly with the increase in temperature, and the curing strength of the resin plugging agent decreases slightly with the increase in ammonium chloride. The results of the comprehensive analysis show that D2 has the best strength after curing, so the dosage of ammonium chloride is determined to be 3%.

##### Optimization of Hexamethylenetetramine Concentration

**Figure 5:**
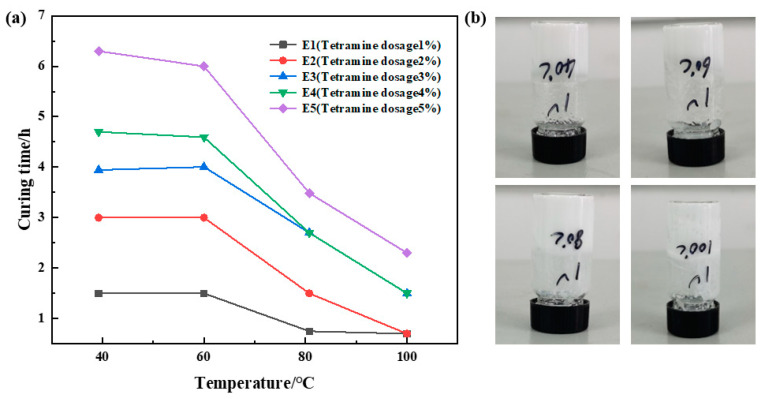
Optimization results of hexamethylenetetramine concentration: (**a**) variation in curing time with temperature at different hexamethylenetetramine dosages; (**b**) curing effect of hexamethylene tetramine at 1% dosage.

The dosage ratio of urea formaldehyde resin was 25%, the dosage ratio of betaine was 1%, the dosage ratio of the silane coupling agent KH-570 was 1%, the dosage ratio of ammonium chloride was 3%, the dosage ratio of hexamethylenetetramine was 1%, 2%, 3%, 4%, and 5%, sodium carboxymethyl cellulose was 1%, and the remainder was deionized water. It was cured at 40 °C, 60 °C, 80 °C, and 100 °C, respectively. Different sample curing temperatures and times were recorded, and the analytical experimental results are shown in Figure 5. The research shows that under the condition of a certain temperature, the more hexamethylenetetramine added, the longer the curing time required. When the hexamethylenetetramine dosage is unchanged, the curing time decreases with the increase in temperature. When the amount of hexamethylenetetramine is greater than 2%, the curing time at a high temperature is gradually extended, and the strength after curing is slightly decreased. The experimental results show that E1 and E2 have good strength after curing. Therefore, the amount of hexamethylenetetramine was determined to be 1%.

##### Optimization of Sodium Carboxymethyl Cellulose Concentration

**Figure 6:**
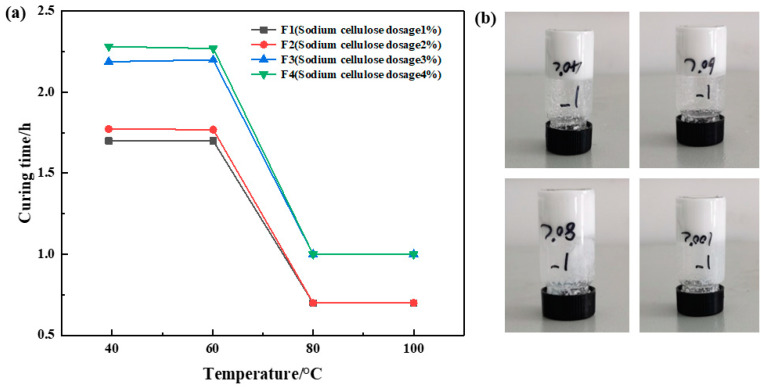
Optimization results of sodium carboxymethyl cellulose concentration: (**a**) variation in curing time with temperature at constant sodium carboxymethylcellulose addition rate; (**b**) curing effect of sodium carboxymethyl cellulose addition at 1%.

The dosage ratio of urea formaldehyde resin was 25%, the dosage ratio of betaine was 1%, the dosage ratio of the silane coupling agent KH-570 was 1%, the dosage ratio of ammonium chloride was 3%, the dosage ratio of hexamethylenetetramine was 1%, the dosage ratio of carboxymethyl cellulose sodium was 1%, 2%, 3%, and 4%, and the remainder was deionized water. It was cured at 40 °C, 60 °C, 80 °C, and 100 °C, respectively. Different sample curing temperatures and times were recorded, and the experimental results are shown in Figure 6. The experimental results showed that the curing time increased slightly with the increase in sodium carboxymethyl cellulose at the same temperature. When the amount of sodium carboxymethyl cellulose is unchanged, the required curing time does not decrease significantly with the increase in temperature, and the more sodium carboxymethyl cellulose added, the lower the curing strength. The comprehensive experimental results show that F1 has the best strength after curing. Therefore, the amount of sodium carboxymethyl cellulose was determined to be 1%.

Through the single factor analysis of the orthogonal test, by changing the dosage of resin, betaine, silane coupling agent KH-570, ammonium chloride, etc., the optimal formula and ratio of resin curing were finally determined as follows: 25% urea formaldehyde resin +1% betaine +1% silane coupling agent KH-570 + 3% ammonium chloride +1% hexamethylenetetramine +1% sodium carboxymethyl cellulose. Optimal curing temperature: 60–80 °C; curing time: 1–3 h.

#### 2.1.2. Structural Characterization of Resin Plugging Agents

##### Optimization of Resin Plugging Agent Formulations

**Figure 7:**
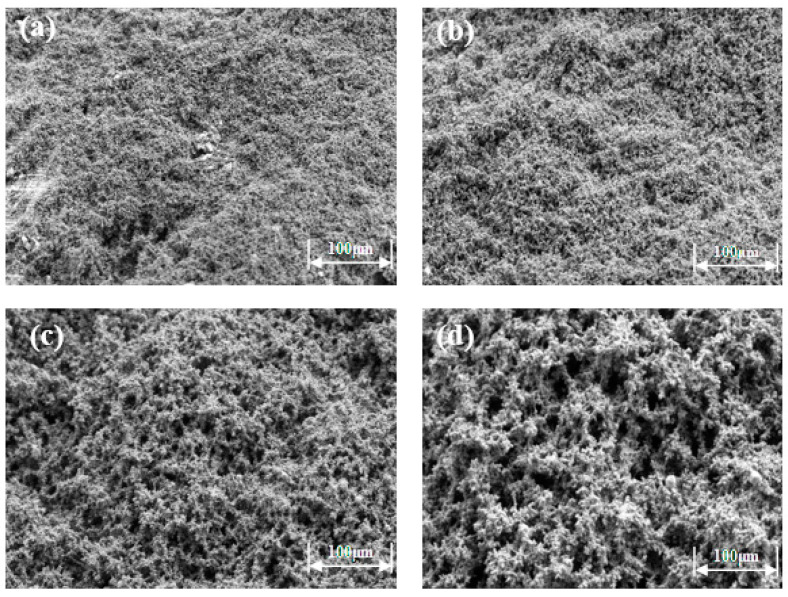
Microstructure of high-strength curable resin at different multiplicities: (**a**) 1000×; (**b**) 2000×; (**c**) 5000×; (**d**) 10,000×.

Scanning electron microscopy was used to observe the microstructure of the prepared high-strength curable resin at different multiples (1000×, 2000×, 5000×, 10,000×), as shown in Figure 7. The high-strength curable resin can form a chain or three-dimensional network structure after curing and cross-linking, and the resin crystal layers are closely connected, which can effectively increase the toughness and strength to a certain extent. The reason for this phenomenon is that in the preparation process, low molecular organosilicon compounds with a special structure are selected as resin cross-linking agents, which contain epoxy, vinyl, amide, alkoxy, and other active functional groups, one end of which can react with inorganic materials, such as glass fibers, silicates, metal oxides, and other surface silyl groups to form covalent bonds. The other end can form a covalent bond with the resin, thus cross-linking the two incompatible materials to form a network structure. At the same time, during curing, active groups, such as hydroxymethyl, amide, and dimethylene ether bonds contained in the resin, will cross-link with formaldehyde to form a three-dimensional spatial network structure.

##### Infrared Analysis

**Figure 8:**
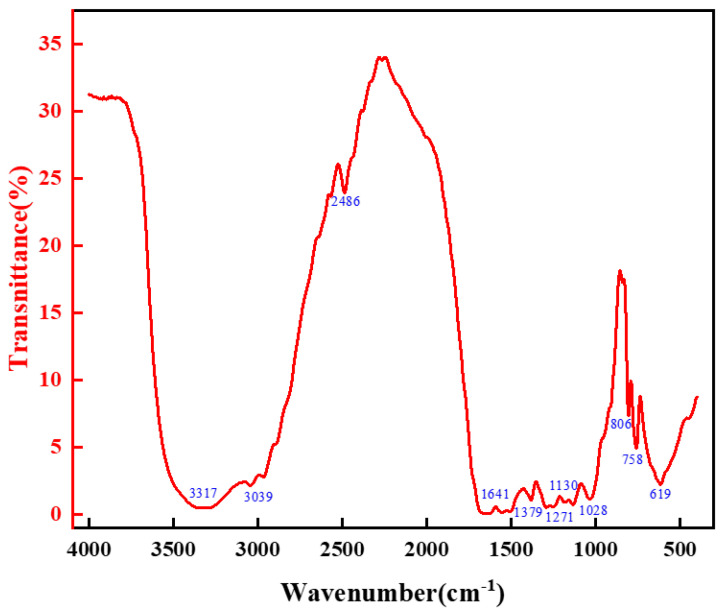
Infrared spectral analysis of resin mortar system.

The chemical structure of the high-strength curable resin was analyzed using a Fourier transform infrared spectrometer (Nicolet iS50 FT-IR, Thermo Fisher Technologies, Shanghai, China). Prior to analysis, the high-strength curable resin was washed with deionized water to remove unreacted components, dried in a vacuum oven, and ground into powder. The samples were prepared using the potassium bromide pressing method, with an infrared spectral scanning range of 4000–400 cm^−1^, at a scanning temperature of 25 °C, a resolution of 1 cm^−1^, and over eight scans. The experimental results are illustrated in Figure 8. The infrared spectroscopic analysis results indicated that the characteristic peaks of the high-strength curable resin changed consistently. The wave number of the amide group peaks gradually shifted to a lower wave number, indicating the formation of more hydroxymethyl and ether bonds. The curing reaction subsequently reduced these structural elements, confirming the successful preparation of the high-strength curable resin.

##### Thermogravimetric Analysis

**Figure 9:**
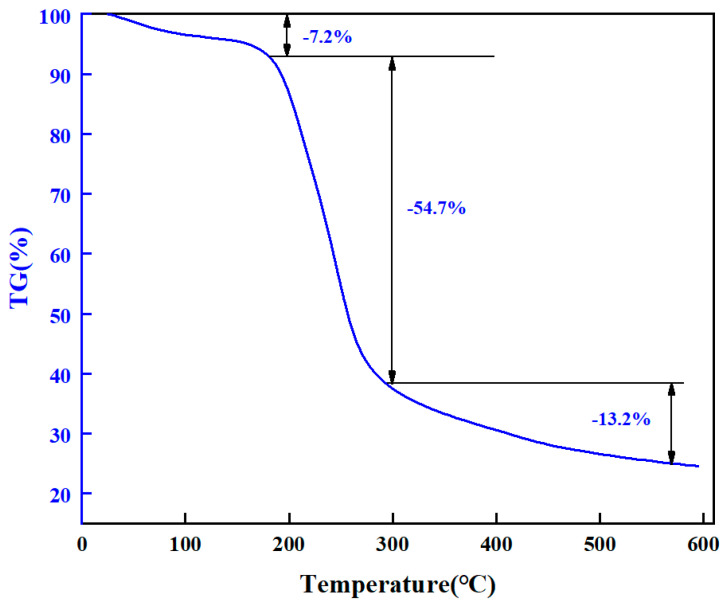
Thermogravimetric analysis of resin system.

The thermogravimetric analyzer (TGA550, Chicago, IL, USA) was used to detect the thermal stability of chemical bonds in high-strength curable resin powders. First, the high-strength curable resin was placed in the oven at 105 °C to remove the water. During each measurement, a sample of 10 to 15 mg of high-strength curable resin was placed in a sealed pan, and the sample was heated from 25 °C to 600 °C at a rate of 20 °C/min. The experiment was carried out in a nitrogen atmosphere of 50 mL/min. The experimental results are shown in Figure 9. Under high-temperature conditions, the curable resin will undergo thermal degradation, causing the molecular chain to break, resulting in the loss of the original properties of the polymer. The thermogravimetric curve of the plugging resin was analyzed to understand its thermal stability. It can be seen from the thermogravimetric curve in Figure 9 that the initial decomposition temperature of the resin system is about 181 °C, and the thermogravimetric weight is divided into three stages. The thermal decomposition of the first stage of the resin begins to occur at about 50 °C, and the weight loss from 50 °C to 181 °C is 7.2%, which is mainly free water and bound water. The weight loss at 295.0 °C is about 54.7%, mainly due to the decomposition of amide groups. At 295.0 °C to 571.0 °C, the thermogravimetric loss reached 13.2%, more hydroxymethyl and ether bonds were formed, and the structural elements were reduced after curing. The results show that the thermal decomposition of the resin plugging agent is stable at about 180 °C, and the thermal stability is good.

### 2.2. Rheological Properties of Resin Plugging Agents

#### 2.2.1. Relationship between Shear Rate and Viscosity

**Figure 10:**
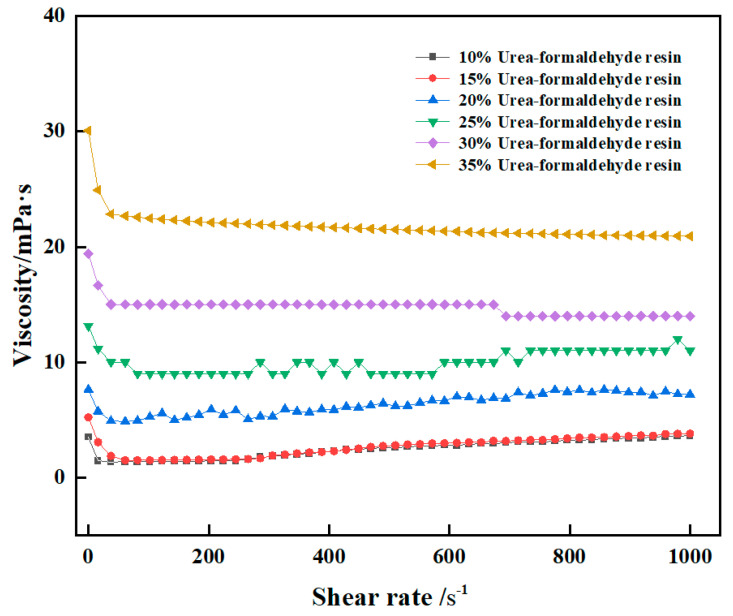
Variation of viscosity with shear rate for high-strength curable resins.

Viscosity is an important parameter to characterize rheological properties. The rheological properties of the high-strength curable resin before curing were tested using the HAAKE MARS 60 high-temperature and high-pressure rotary rheometer. The viscosity of the high-strength curable resin solution before curing changed with the shear rate measured by the Harker rheometer, and the results are shown in Figure 10. The experimental results show that the viscosity of the high-strength curable resin system can stay stable with the increase in shear rate. The viscosity of the curable resin system with the addition of 10–15% resin matrix is about 2–3 mPa·s, and the viscosity is low. The viscosity of the curable resin system with the addition of 20–30% resin matrix is about 5–15 mPa·s, and the viscosity is moderate. The viscosity of the curable resin system with the addition of 35% resin matrix is about 22 mPa·s. When the shear rate is less than 100 s^−1^, the viscosity gradually decreases and becomes stable. When the shear rate is greater than 100 s^−1^, the viscosity remains basically unchanged. When 10% resin was added, the viscosity was stable at 2 mPa·s. The viscosity is stable at 7 mPa·s when 20% resin is added. When 30% resin is added, the viscosity is stable at 15 mPa·s. The viscosity is stable at 22 mPa·s when 35% resin is added. Overall, in order to facilitate the injection of a high-strength curable resin system and its mixing with suspension materials, the resin system should have a certain viscosity to facilitate the suspension of other additive materials.

#### 2.2.2. Relationship between Shear Stress and Modulus

**Figure 11:**
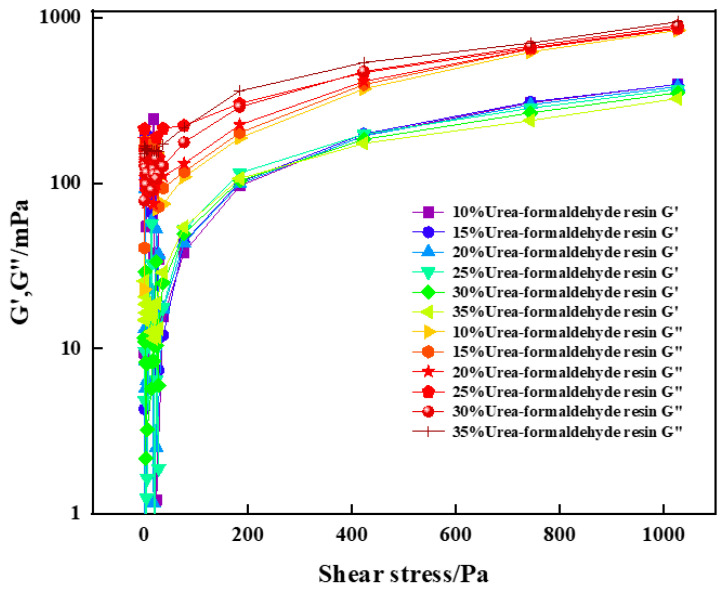
Variation of energy storage modulus and loss modulus with shear stress for high-temperature-resistant, high-strength curable resins.

The energy storage modulus (G′) and the loss modulus (G″) are important parameters for characterizing rheological properties. The changes of the energy storage modulus and loss modulus with shear stress before curing of high-strength curable resin solution with different concentrations were tested by the HAAKE MARS 60 high temperature and high pressure rotational rheometer. The experimental results are shown in Figure 11. The experimental results show that the energy storage modulus and loss modulus of the cured resin solution with different concentration and high strength increase with the increase of shear stress, and the loss modulus is greater than the energy storage modulus, which is a viscous fluid. At the same time, it is found that the increase of urea-formaldehyde resin content has little effect on the energy storage modulus and loss modulus of the curable resin, and the change law is basically consistent with the increase of shear stress.

### 2.3. Curing Properties of High Strength Curable Resin Plugging Agent

#### 2.3.1. Effect of Temperature on the Curing Effect of Resin Plugging Agent

The resin was formulated using the optimal proportions of high-strength curable resin: 25% urea-formaldehyde resin, 1% betaine, 1% silane coupling agent KH-570, 3% ammonium chloride, 1% hexamethylene tetramine, and 1% sodium carboxymethyl cellulose. The prepared high-strength curable resin plugging agent was cured at 40 °C, 60 °C, 80 °C, and 100 °C to observe the curing time and strength. The experimental results are presented in Table 1. The research shows that the resin plugging agent prepared according to the optimal formulation and proportion of the high strength curable resin can be cured at 40–100 °C, and the curing strength is good. It was also found that the curing time of high strength curable resin decreased with the increasing of curing temperature. At 40 °C, the resin plugging agent needs 2.6 h to achieve strong curing strength. The curing time is 1 h at 60 °C. When the temperature rises to 100 °C, the curing time is only 0.67 h, and there are more pores in the cured resin plugging agent, and its curing strength is slightly weakened. The experimental results show that the resin plugging agent has the best curing effect at 60~80 °C.

#### 2.3.2. Effect of Salt Ion Concentration on the Curing Effect of Resin Plugging Agents

Since resin plugging agent will be eroded by formation water in the formation, prediction is very important for the anti-erosion ability of different salt solutions. In this study, sodium ion solution and calcium ion solution of different concentrations were selected to simulate formation water, and the influence of salinity on the curing effect of resin plugging agent was studied.

##### Effect of Sodium Ion Concentration on the Curing Effect of Resin Plugging Agent

**Figure 12:**
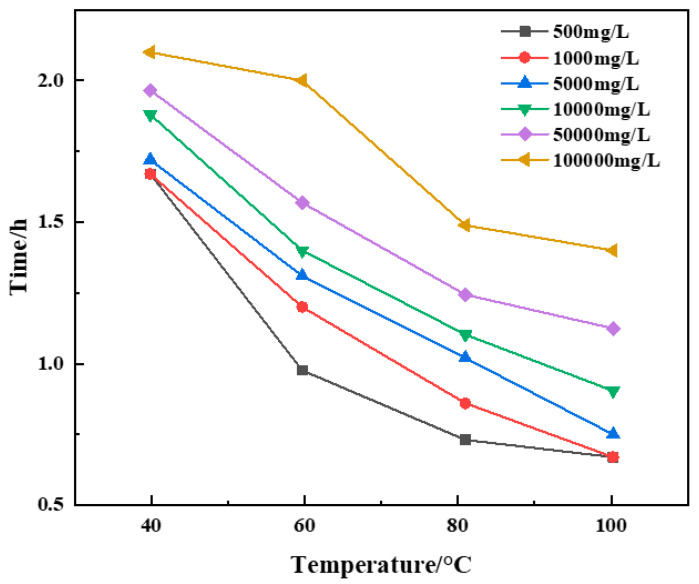
Variation of curing time with temperature at constant sodium ion solution concentration.

Aqueous solutions containing Na^+^ were prepared with concentrations of 500 mg/L, 1000 mg/L, 5000 mg/L, 10,000 mg/L, 50,000 mg/L and 100,000 mg/L respectively, and resin plugging systems were prepared with different concentrations of salt water. The influence of salt water concentrations on the curing effect of resin plugging agent at different temperatures was studied. The experimental results are shown in Figure 12 and Table 2.

**Figure 13:**
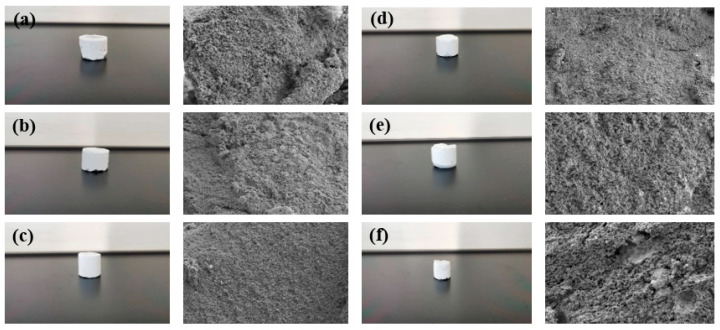
Controllable curing resin and microstructure prepared under different salinity conditions: (**a**) 500 mg/L; (**b**) 1000 mg/L; (**c**) 5000 mg/L; (**d**) 10,000 mg/L; (**e**) 50,000 mg/L; (**f**) 100,000 mg/L.

The experimental results show that saline with different concentrations of Na^+^ can prolong the curing time of a resin plugging system, and saline with different concentrations of Na^+^ can enhance the curing strength of a resin plugging system under certain temperature conditions. The higher the concentration, the harder the strength. The consolidated body and its microstructure obtained after curing of the resin plugging system prepared with salt water of different concentrations of Na^+^ are shown in Figure 13. The results show that when the concentration of Na+ was between 500 mg/L and 10,000 mg/L, with the increase in Na^+^ concentration, the surface became smoother and denser, and the three-dimensional network structure formed by the connection became more uniform. However, the curing strength was slightly weaker when the concentration continued to increase and the temperature was higher. At 60 °C, the curing strength of the resin increased from 5.22 MPa to 5.75 MPa with the increase in Na^+^ concentration. At 80 °C, the curing strength of the resin increased from 5.31 MPa to 5.87 MPa with the increase in Na^+^ concentration. At 100 °C, the curing strength of the resin increased from 4.85 MPa to 5.68 MPa with the increase in Na^+^ concentration.

**Figure 14:**
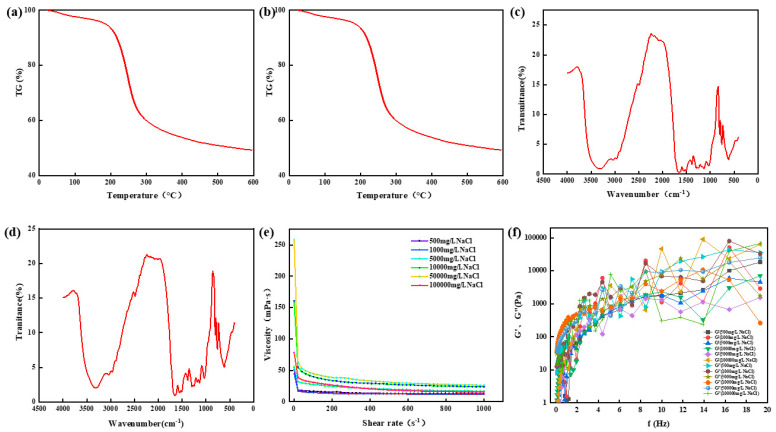
Physicochemical properties of thermosetting resin sealers. (**a**) Thermogravimetric analysis of samples with Na+ concentration of 5000 mg/L. (**b**) Thermogravimetric analysis of samples with Na+ concentration of 10,000 mg/L. (**c**) Infrared spectrum analysis of samples with Na+ concentration of 5000 mg/L. (**d**) Infrared spectrum analysis of samples with Na+ concentration of 5000 mg/L. (**e**) Viscosity change of resin system under different Na^+^ concentrations. (**f**) The change in the energy storage modulus and the loss modulus of the resin system with frequency.

The thermogravimetric analyzer (TGA550, Chicago, IL, USA) was used to determine the thermal stability of chemical bonds in high-strength curable resin powders prepared at different Na^+^ concentrations. First, the high-strength curable resin was placed in the oven at 105 °C to remove the water. During each measurement, a sample of 10 to 15 mg of high-temperature-resistant, high-strength curable resin was placed in a sealed pan, and the sample was heated from 25 °C to 600 °C at a rate of 20 °C/min. The experiment was carried out in a nitrogen atmosphere of 50 mL/min. The experimental results are shown in Figure 14a,b. It can be seen from observation that when the Na^+^ concentration is 50,000 mg/L and 100,000 mg/L, the initial decomposition temperature of the resin system is about 200 °C, which is higher than that of the resin system prepared with deionized water, and it has good thermal stability.

The chemical structure of high-strength curable resin was measured through Fourier transform infrared spectroscopy (Nicolet iS50 FT-IR, Thermo Fisher Technologies, Shanghai, China). Before testing, the high-strength curable resin was cleaned with deionized water to remove the unreacted part of the curable resin, and then the resin was dried in a vacuum oven and ground into a powder. The sample was prepared through the potassium bromide tablet method. The scanning range of the infrared spectrum was 4000–400 cm^−1^, the scanning temperature was 25 °C, the resolution was 1 cm^−1^, and the scanning times were eight times. The experimental results are shown in Figure 14c,d. The experimental results show that after the curing of the resin system with Na^+^ concentrations of 50,000 mg/L and 100,000 mg/L, the structural elements, such as hydroxymethyl and ether bonds, gradually decrease, and a new high-strength consolidated body is formed.

Viscosity, energy storage modulus (G′), and loss modulus (G″) are important parameters for characterizing rheological properties. The rheological properties of the high-strength curable resin before curing were tested using the HAAKE MARS 60 high-temperature and high-pressure rotary rheometer. The viscosity of the high-strength curable resin solution prepared with different Na^+^ concentrations tested using the Harker rheometer changed with the shear rate before curing. The experimental results are shown in Figure 14e. The results show that the viscosity of the resin system gradually decreases with the increase in the shear rate and finally tends to be stable, with the viscosity ranging from 16 to 40 mPa·s, indicating that the resin system is a viscous fluid. At the same time, it was found that the higher the concentration of Na^+^, the higher the viscosity of the prepared high-temperature and high-strength curable resin plugging agent system. Figure 14f shows the changes in the energy storage modulus and loss modulus of the resin system with different Na^+^ concentrations with frequency. The experimental results show that the energy storage modulus and loss modulus increase with the increase in shear frequency, and the energy storage modulus is always smaller than the loss modulus, indicating that the resin system has good viscosity characteristics.

##### Effect of Calcium Ion Concentration on the Curing Effect of Resin Plugging Agents

**Figure 15:**
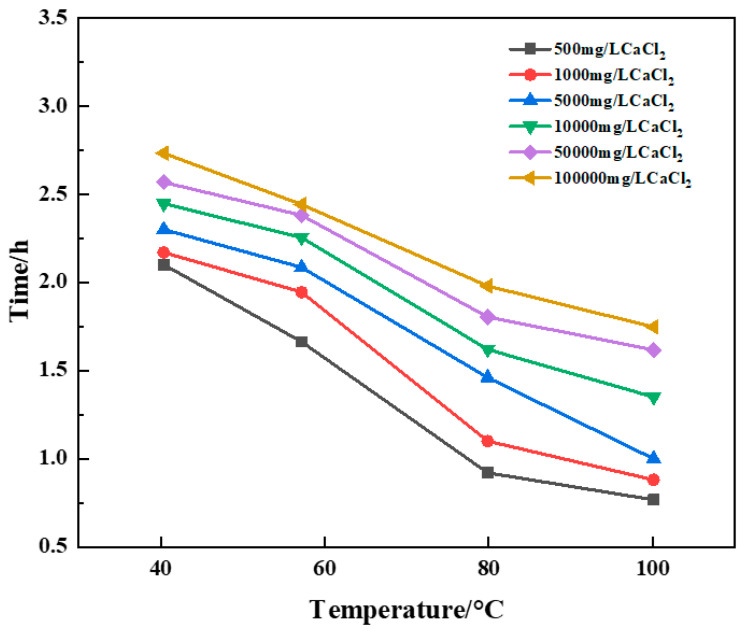
Variation of curing time with temperature for constant Ca^2+^ brine concentration.

Aqueous solutions containing Ca^2+^ were prepared with concentrations of 500 mg/L, 1000 mg/L, 5000 mg/L, 10,000 mg/L, 50,000 mg/L, and 100,000 mg/L, respectively, and resin plugging systems were prepared with different concentrations of saline. The effects of saline concentrations on curing time at different temperatures were studied. The experimental results are shown in Figure 15 and Table 3.

**Figure 16:**
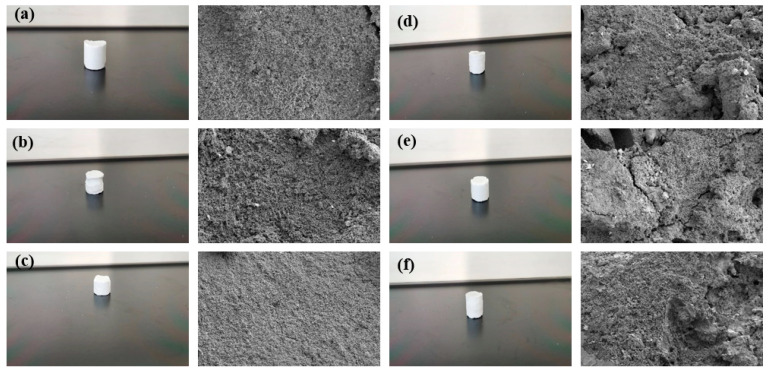
The sample and its microstructure were cured using a resin plugging system with different Ca^2+^ concentrations: (**a**) 500 mg/L; (**b**) 1000 mg/L; (**c**) 5000 mg/L; (**d**) 10,000 mg/L; (**e**) 50,000 mg/L; (**f**) 100,000 mg/L.

The experimental results showed that the curing time of the resin plugging system was increased, and the curing strength of the resin was greatly affected by the preparation of a resin plugging slurry with salt water containing different concentrations of Ca^2+^. The higher the temperature, the shorter the curing time of the resin plugging system. At the same time, different concentrations of Ca^2+^ brine have a great influence on the curing strength of resin, and the curing strength of the resin plugging system prepared with 5000 mg/L~100,000 mg/LCa^2+^ brine is the best. The resin samples prepared with Ca^2+^ brine of different concentrations were tested through scanning electron microscopy to observe their microstructure, and the results are shown in Figure 16. Through observation, it can be seen that when the concentration of Ca^2+^ is between 50,000 mg/L and 100,000 mg/L, the three-dimensional network structure formed is dense and uniform. When the concentration decreases and the temperature is high, the curing strength is weak, and the curing time is generally slightly longer than that of the resin mortar prepared with Na^+^ brine, which has a greater impact on the curing strength of the resin and reduces the curing strength of the resin plugging system to a certain extent.

**Figure 17:**
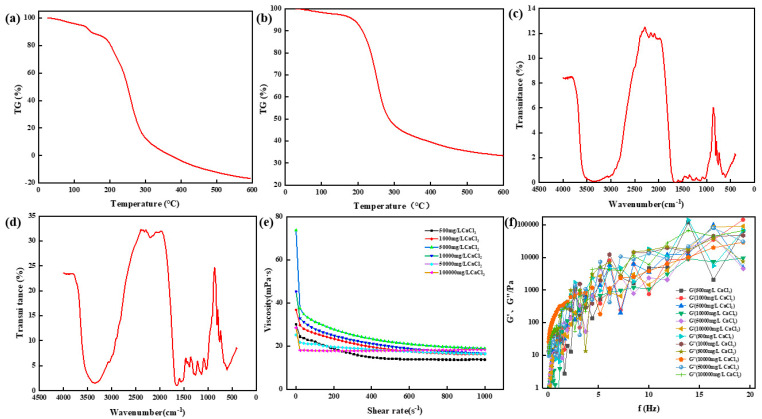
Physicochemical properties of thermosetting resin sealers. (**a**) Thermogravimetric analysis of samples with Ca^2+^ concentration of 5000 mg/L. (**b**) Thermogravimetric analysis of samples with Ca^2+^ concentration of 10,000 mg/L. (**c**) Infrared spectrum analysis of samples with Ca^2+^ concentration of 5000 mg/L. (**d**) Infrared spectrum analysis of samples with Ca^2+^ concentration of 5000 mg/L. (**e**) Viscosity change of resin system under different Ca^2+^concentrations. (**f**) The change in the energy storage modulus and the loss modulus of the resin system with frequency.

The thermogravimetric analyzer (TGA550, Chicago, IL, USA) was used to determine the thermal stability of chemical bonds in high-temperature-resistant, high-strength curable resin powders prepared at different Ca^2+^ concentrations. The high-strength curable resin was placed in an oven at 105 °C to remove the water. The experiment was carried out in a nitrogen atmosphere of 50 mL/min; each time, 10~15 mg of the high-temperature-resistant, high-strength curable resin sample was put into a sealed pan, and the sample was heated from 25 °C to 600 °C at a rate of 20 °C/min. The experimental results are shown in Figure 17a,b. Through observation, it can be concluded that the initial decomposition temperature of the resin system prepared with a Ca^2+^ concentration of 50,000 mg/L and 100,000 mg/L is about 200 °C, the thermal weight loss mainly has two stages, and the thermal stability is good.

The chemical structure of the high-strength curable resin was tested through Fourier transform infrared spectroscopy (Nicolet iS50 FT-IR). Before the test, the high-temperature-resistant, high-strength curable resin was cleaned with deionized water to remove the unreacted part of the high-temperature-resistant, high-strength curable resin, and then the high-strength curable resin was dried in a vacuum oven and ground into a powder. The sample was prepared using the potassium bromide tablet method. The scanning range of the infrared spectrum was 4000–400 cm^−1^, the scanning temperature was 25 °C, the resolution was 1 cm^−1^, and the scanning times were eight times. The experimental results are shown in Figure 17c,d. The results show that the infrared spectra of the resin system prepared with brine of different Ca^2+^ concentrations are not much different from those prepared with deionized water, and the relevant functional group structure can still be guaranteed.

The viscosity of a high-strength curable resin solution prepared with brine of different Ca^2+^ concentrations was measured with the shear rate before curing using a HAAKE MARS 60 rotational rheometer. The experimental results are shown in Figure 17e. The results show that the viscosity of the resin system gradually decreases with the increase in the shear rate and finally tends to be stable, with the viscosity between 16 and 24 mPa·s. The changes in the energy storage modulus and loss modulus of the resin system with frequency in brine with different Ca^2+^ concentrations are shown in Figure 17f. Both the energy storage modulus and loss modulus increase with the increase in frequency, and the energy storage modulus is always smaller than the loss modulus, indicating that the resin system presents good viscosity characteristics.

### 2.4. Plugging Properties of High-Strength Curable Resin Plugging Agent

The high-strength curable resin solution is injected into the lost formation and stays in the fracture space. Under the action of the formation’s temperature, a cross-linked, high-strength, consolidated body is formed to prevent further loss of drilling fluid. In order to study the plugging rule of the high-strength curable resin system in fractures, a high-temperature and high-pressure plugging and displacement device was used in the experiment. The high-strength curable resin system was injected into core fractures of different inlet and outlet sizes at a certain injection speed for aging, and then displacements were performed after curing to evaluate the plugging effect of high-strength curable resin under different fracture conditions.

#### 2.4.1. Pressure-Plugging Ability of Resin Plugging System in Parallel Fractures

**Figure 18:**
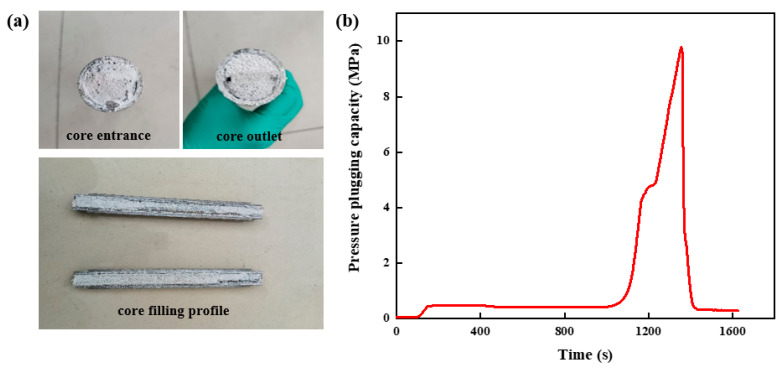
Pressure-plugging ability of resin plugging system in parallel fractures. (**a**) Parallel fracture plugging and replacement core filling effect. (**b**) Pressure-plugging effect of parallel fractures.

The high-strength curable resin plugging agent solution was prepared and injected into the high-temperature and high-pressure plugging and displacement experimental device. After it was cured in the fracture’s core, it was taken out. The slug shape was formed in the parallel fracture with an inlet and outlet size of 3 mm, as shown in Figure 18a. It can be seen that the high-strength curable resin system can achieve complete filling in the fracture, and, after curing, the overall integrity is high, the gravity settlement effect is weak, and it can achieve uniform distribution in the fracture, forming a consolidated body with high toughness, realizing the effective plugging of the fracture. The solidified core was used for the pressure test, and its pressure change curve is shown in Figure 18b. With the continuous injection of fluid, the pressure of the high-strength curable resin system in the parallel fractures with inlet and outlet sizes of 3 mm gradually increased, and when the pressure reached 9.78 MPa, the pressure broke through and dropped sharply. This shows that the structure of the high-strength curable resin has deformed and fractured under the action of fluid pressure. At the same time, the resin plugging system gradually began to discharge from the core outlet, and the internal pressure of the core decreased rapidly.

#### 2.4.2. Pressure-Plugging Ability of Resin Plugging System in Wedge Fractures

##### Wedge Fracture Entrance 5 mm, Exit 3 mm

**Figure 19:**
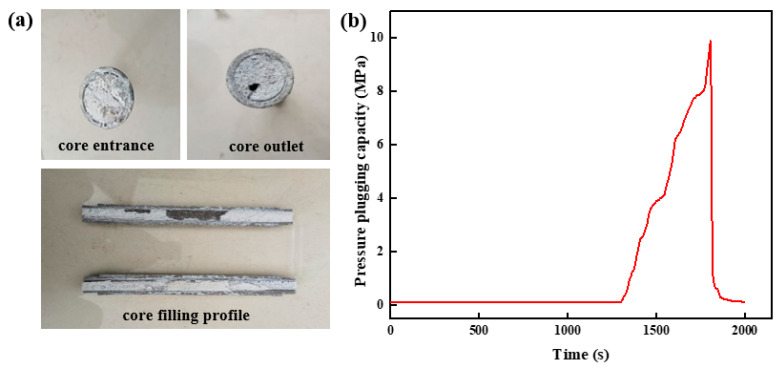
Pressure-plugging ability of resin plugging system in wedge fracture (5 mm inlet, 3 mm outlet). (**a**) Wedge fracture core-filling effect. (**b**) Pressure-plugging effect of wedge fracture.

The shape of the high-strength curable resin plugging system solution after curing in the wedge fracture with an inlet end of 5 mm and an outlet end of 3 mm to form a slug is shown in Figure 19a. The high-strength curable resin solidifies after polymerization reaction and has a weak gravitational settling effect. It can fill wedge fractures evenly and effectively plug fractures. This is mainly due to the high-strength curable resin solution’s viscosity; the gravity settlement effect is small, and it easily stays in the fractured formation to form a uniform and effective filling. The high-strength curable resin system was injected into the plugging test device according to the plugging test steps, and the whole plugging layer was formed after solidification and then displaced. The pressure change curve inside of the core was obtained, as shown in Figure 19b. When the pressure reaches 9.98 MPa, the pressure drops instantaneously, indicating that the integrity of the resin plugging layer has been destroyed under the action of fluid pressure. After breaking through the resin barrier, the pressure drops rapidly and becomes stable.

##### Wedge Fracture Entrance 12 mm, Exit 10 mm

**Figure 20:**
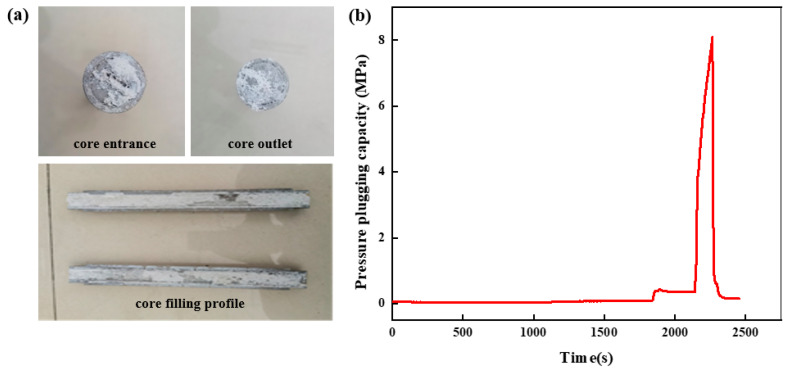
Pressure-plugging ability of resin plugging system in wedge fracture (12 mm inlet, 10 mm outlet). (**a**) Wedge fracture core-filling effect. (**b**) Pressure-plugging effect of wedge fracture.

The high-strength curable resin solution was prepared and injected into the high-temperature and high-pressure plugging experimental device. The plugging effect after curing in the wedge fracture with an inlet end size of 12 mm and an outlet end size of 10 mm to form a slug is shown in Figure 20a. As it can be seen from Figure 20a, the resin plugging system has achieved complete filling in the wedge fracture, and the resin has good overall integrity, can be evenly distributed in the fracture, fills the fracture more fully, polymerizes to form a resin plugging layer under certain temperature conditions, and effectively plugs the fracture. The breakthrough pressure curve of the high-strength curable resin plugging system is shown in Figure 20b. In the early stage of the experiment, the change in pressure is not obvious, while the change in pressure is obvious near the maximum pressure, and it will soon break the maximum pressure and decrease rapidly. Then, the pressure tends to be stable. The high-strength curable resin plugging system can withstand the maximum pressure of 8.1 MPa in the wedge fracture of 12 mm at the entrance and 10 mm at the exit. With the increase in the fracture width, the maximum pressure of the resin plugging agent decreases gradually.

In summary, it can be seen that the high-strength curable resin plugging system can effectively reside in different fracture types (parallel, wedge) and different fracture sizes and form a high-strength consolidated body under certain temperature conditions for effective plugging. Under the same outlet size, the breakthrough pressure of the resin plugging system in the wedge fracture is greater than that in the parallel fracture. The main reason is that the formation pressure and viscous resistance of the resin plugging system in the wedge fracture are opposite to the fluid impact pressure, which can effectively slow down the erosion of the resin plugging system and increase the maximum breakthrough pressure. In parallel fractures, only the viscous resistance resists the fluid impact pressure, and the breakthrough pressure is relatively reduced. Meanwhile, it is found that the smaller the fracture size of the outlet end, the greater the breakthrough pressure of the resin plugging agent system, and the better the plugging effect. The breakthrough pressure of the high-strength curable resin plugging system can reach 8.1 MPa in the wedge fracture with a fracture width of 10 mm. The smaller the fracture width, the greater the breakthrough pressure. In the wedge fracture with a fracture width of 3 mm at the exit, the breakthrough pressure of the high-strength curable resin plugging system reaches 9.98 MPa, which can form a plugging layer with high strength.

## 3. Conclusions

(1) Through the single-factor analysis of the orthogonal test, changing the dosage of resin, betaine, silane coupling agent KH-570, ammonium chloride, etc., the optimal formula and ratio of resin curing were finally determined as follows: 25% urea formaldehyde resin +1% betaine +1% silane coupling agent KH-570 + 3% ammonium chloride +1% hexamethylenetetramine +1% sodium carboxymethyl cellulose. The prepared resin plugging agent has the best curing effect at 60~80 °C, and the curing time is controllable from 1 to 3 h.

(2) The prepared high-strength curable resin can form a three-dimensional network structure after curing and cross-linking, and the resin crystal layers are closely connected, which can effectively increase the toughness and strength to a certain extent. Meanwhile, the thermal decomposition of the consolidated body of the resin plugging system is stable at about 180 °C, which has good thermal stability.

(3) The experimental results show that the viscosity of the high-strength curable resin system can keep stable with the increase in the shear rate. The viscosity of the curable resin system with the addition of 10–15% resin matrix is about 2–3 mPa·s, and the viscosity is low. The viscosity of the curable resin system with the addition of 20–30% resin matrix is about 5–15 mPa·s, and the viscosity is moderate. The viscosity of the curable resin system with the addition of 35% resin matrix is about 22 mPa·s. Additionally, the energy storage modulus and loss modulus of various concentrations of high-strength curable resin solutions before curing augmented with escalating shear stress, where the loss modulus surpassed the energy storage modulus, reflecting a viscous fluid behavior.

(4) The formation water containing Na^+^ increased the curing strength of the resin and reduced the curing time between 500 mg/L and 10,000 mg/L. Too high a concentration will reduce the curing strength at lower temperatures. The formation water containing Ca^2+^ will increase the curing time of the resin plugging system, and it has a great influence on the curing strength of the resin plugging system, reducing the curing strength of the resin plugging system to a certain extent.

(5) The high-strength curable resin plugging system can effectively reside in different fracture types (parallel, wedge) and different fracture sizes and form a high-strength consolidated body under certain temperature conditions for effective plugging. The breakthrough pressure of the high-strength curable resin plugging agent system can reach 8.1 MPa in the wedge fracture with a fracture width of 10 mm. The smaller the fracture width, the greater the breakthrough pressure. In the wedge fracture with a fracture width of 3 mm at the exit, the breakthrough pressure of the high-strength curable resin plugging system reaches 9.98 MPa, which can form a plugging layer with high strength.

## 4. Experimental Section

### 4.1. Chemical Substance

Urea formaldehyde resin (UF, 99%), epoxy resin (ER, 98%), all analytical grade, Aladdin Chemical Reagent Co., Ltd. (Shanghai, China); betaine (96%), hexamethylenetetramine (96%), phenolic resin (99%), all analytical grade, Shanghai McLean Biochemistry Co., Ltd. (Shanghai, China); sodium carboxymethyl cellulose (99%), ammonium chloride (99%), silane coupling agent (KH-570, 99%), barite (99.8%), and potassium persulfate (KPS, 99.9%), all analytical grade, Sinopharm Chemical Reagent Co., Ltd. (Shanghai, China).

### 4.2. Preparation Method of Resin Plugging Agent

Add 163 g of formaldehyde and 60 g of urea into a three-necked flask, adjust the pH to 7.8–8.0, and then raise the temperature to about 90 °C in 30–40 min and keep warm for 35 min. After the holding time was completed, the pH was adjusted to 4.0–4.5, and when a turbidity point appeared at this temperature, the pH was immediately adjusted to 7.0–7.5, and then the second batch of urea 15 g was added, and the holding time was 30 min at this temperature. At the end of the holding time, add the third batch of urea 25 g, hold for 30 min, adjust the pH to 8.0–9.0, cool down, and remove. The resin’s final pH = 8.9, the solid content is 53%, and the initial viscosity is 35 mPa·s. Based on the method of physical blending and chemical modification, the resin material is selected as the matrix, and the curing agent, flow regulator, cross-linking agent, and aggravating material are added and stirred in deionized water to form the resin plugging agent. The cross-linking and curing reaction can take place at a certain temperature to produce a high-strength solidified body. The addition of resin plugging agent components is shown in Table 4.

### 4.3. Rheology

The rheological properties of the samples before curing of the resin plugging agent system were tested using a HAAKE MARS 60 rotational rheometer. The rotor model used for the experiments was CC41 /Ti (rotor diameter 41 mm). The temperature of the test samples was equilibrated for at least 30 min, and the temperature error was controlled to ±0.1 °C. The temperature was controlled to ±0.1 °C. The apparent viscosity, energy storage modulus (G’), and loss modulus (G”) of the resin plugging agent samples in the linear viscoelastic region were measured. The strain range was γ = 100%, and the frequency variation range was 1 Hz. To ensure the accuracy of the data, the above rheological property tests were repeated three times.

### 4.4. Infrared Spectrum

The chemical structure of the resin plugging agent was analyzed using Fourier transform infrared spectroscopy (Nicolet iS50 FT-IR). Prior to analysis, the cured resin plug samples underwent a deionized water wash to eliminate any unreacted components. Subsequently, the samples were dried in a vacuum oven and pulverized into powder form. The samples were prepared using the potassium bromide pressing method, and the infrared spectra were acquired within the range of 4000–400 cm^−1^ at a scanning temperature of 25 °C. Spectra were collected with a resolution of 1 cm^−1^ and an average of 8 scans.

### 4.5. Thermogravimetric Analysis

Thermal stability of the chemical bonds in the resin plugging agent powder was assessed using a thermogravimetric analyzer (TGA550, Chicago, IL, USA). Initially, the resin plugging agent underwent dehydration in an oven at 105 °C. For each measurement, a sample weighing 10 to 15 mg of the resin plugging agent was enclosed in a sealed pan and subjected to heating from 25 °C to 600 °C at a rate of 10 °C/min. The experiments were conducted under a nitrogen atmosphere at a flow rate of 50 mL/min.

### 4.6. Scanning Electron Microscope (SEM)

The microstructure of the resin plugging agent samples was characterized using a Hitachi S-4700 field emission scanning electron microscope (SEM). The treated resin plug samples underwent careful sectioning to obtain a fresh cross-section. Subsequently, they were mounted on aluminum stubs, coated with a thin layer of gold for enhanced conductivity, and subjected to SEM imaging at 10 kV.

### 4.7. Compressive Strength

Urea formaldehyde resin plugging material was cured into a cylindrical shape with a bottom diameter of 10 mm and a height of 10 mm. Compression mechanical properties were tested using an electronic universal testing machine (CMT4000 electronic universal testing machine, Shenzhen New Sansi materials testing company, Shenzhen, China) at room temperature, with the compression speed set to 3 mm/min. The resulting compression stress–strain curve of the resin samples was recorded.

### 4.8. Plugging Performance

A physical simulator designed to replicate high-temperature and high-pressure conditions was employed to investigate the plugging efficacy of curable resins on fractures. The simulated fracture cores, resembling steel columns, featured longitudinal fractures with dimensions including a length of 30 cm, a height of 3 cm, and widths of 3, 5, 7, and 10 mm, respectively. The plugging test procedure comprised several steps: (a) adjusting the temperature of the heating box to 80 °C to match simulated stratigraphic conditions; (b) placing steel fracture cores of various seam widths into core grippers under a peripheral pressure of 10 MPa; (c) injecting simulated drilling fluids into the fracture cores at a rate of 10.0 mL/min until saturation; (d) injecting curable resin solution into the fractured cores at a rate of 10.0 mL/min until the fracture outlet emits only resin solution without water; (e) plugging the fractured core model and allowing 8 h for the resin solution reaction to complete; (f) injecting the resin solution into the fractured cores at a rate of 10.0 mL/min until the resin reaction is completed; (g) injecting the resin solution into the fractured cores at 80 °C; and (h) reversing the injection of simulated drilling fluid into the fractured cores at a rate of 10.0 mL/min, while using data software to monitor real-time changes in injection pressure. The highest pressure recorded represents the resin’s pressure-bearing plugging capacity on the fracture.

## Figures and Tables

**Table 1:** Curing time and strength of resin plugging agent samples at different temperatures.

Sample NameY-1	40 °C	60 °C	80 °C	100 °C
Curing time	2.6 h	1 h	0.67 h	0.67 h
Curing Strength	3.78 MPa	5.22 MPa	5.31 MPa	4.85 MPa

**Table 2:** Effect of different sodium ion solution concentrations on the cured strength of resin plugging agent.

Na^+^ Solution	500 mg/L	1000 mg/L	5000 mg/L	10,000 mg/L	50,000 mg/L	100,000 mg/L
40 °C	3.78 MPa	4.07 MPa	3.25 MPa	3.37 MPa	4.14 MPa	4.59 MPa
60 °C	5.22 MPa	5.28 MPa	5.33 MPa	5.75 MPa	5.49 MPa	5.21 MPa
80 °C	5.31 MPa	5.44 MPa	5.51 MPa	5.87 MPa	5.75 MPa	5.32 MPa
100 °C	4.85 MPa	4.95 MPa	5.36 MPa	5.68 MPa	5.11 MPa	5.04 MPa

**Table 3:** Effect of different Ca^2+^ brine concentrations on the cured strength of resin plugging agent.

Ca^2+^ Solution	500 mg/L	1000 mg/L	5000 mg/L	10,000 mg/L	50,000 mg/L	100,000 mg/L
40 °C	3.65 MPa	3.88 MPa	3.57 MPa	3.77 MPa	4.14 MPa	4.39 MPa
60 °C	4.22 MPa	4.78 MPa	5.33 MPa	5.75 MPa	5.49 MPa	5.21 MPa
80 °C	4.31 MPa	4.49 MPa	5.31 MPa	5.67 MPa	5.95 MPa	5.88 MPa
100 °C	4.49 MPa	4.60 MPa	4.95 MPa	5.38 MPa	5.78 MPa	5.70 MPa

**Table 4:** Resin plugging agent component dosage.

Constituent	Urea Formaldehyde Resin	Betaine	Silane Coupling Agent	Ammonium Chloride	Hexamethylenetetramine	Sodium Carboxymethyl Cellulose
Concentration/%	25	1	1	3	1	1

## Data Availability

The data presented in this study are openly available in article.
